# Use of complementary and alternative medicine in Norway: a cross-sectional survey with a modified Norwegian version of the international questionnaire to measure use of complementary and alternative medicine (I-CAM-QN)

**DOI:** 10.1186/s12906-021-03258-6

**Published:** 2021-03-16

**Authors:** Agnete Egilsdatter Kristoffersen, Sara A. Quandt, Trine Stub

**Affiliations:** 1 Department of Community Medicine, National Research Center in Complementary and Alternative Medicine (NAFKAM), UiT The Arctic University of Norway, N-9037 Tromsø, Norway; 2 Department of Epidemiology and Prevention Division of Public Health Sciences Wake Forest School of Medicine Medical Center Boulevard Winston-Salem, Winston-Salem, North Carolina 27157-1063 USA

## Abstract

**Background:**

In recent decades complementary and alternative medicine (CAM) has been widely used worldwide as well as in Norway, where CAM is offered mainly outside the national health care service, mostly complementary to conventional treatment and fully paid for by the patients. With few exceptions, previous research has reported on frequency and associations of total CAM use in Norway rather than on single therapies and products. Therefore, in this present study we will map the use of CAM more precisely, including types of services, products, and self-help practices and further include reasons for use and helpfulness of the specific therapies used based on a modified Norwegian version of the I-CAM-Q (I-CAM-QN).

**Method:**

Computer assisted telephone interviews using I-CAM-QN were conducted with 2001 randomly selected Norwegians aged 16 and above using multistage sampling in January 2019 with age and sex quotas for each area. Weights based on sex, age, education, and region corrected for selection biases, so that results are broadly representative of the Norwegian population. Descriptive statistics were carried out using Pearson’s Chi-square tests and t-tests to identify group differences.

**Result:**

CAM use was reported by 62.2% of the participants during the prior12 months. Most participants had used natural remedies (47.4%), followed by self-help practices (29.1%) and therapies received from CAM providers (14.7%). Few of the participants had received CAM therapies from physicians (1.2%). Women were generally more likely to use CAM than men, younger people more likely than older, and participants with lower university education and income more likely than participants without university education, with higher university education and higher income. Mean number of visits per year to the different CAM providers ranged from 3.57 times to herbalists to 6.77 times to healers. Most of the participants found their use of CAM helpful.

**Conclusion:**

This study confirms that CAM is used by a considerable segment of the Norwegian population. We suspect that the number of participants reporting CAM use is greater when specific therapies are listed in the questionnaire as a reminder (as in the I-CAM-QN) compared to more general questions about CAM use. The CAM modalities used are mainly received from CAM providers operating outside public health care or administered by the participants themselves.

**Supplementary Information:**

The online version contains supplementary material available at 10.1186/s12906-021-03258-6.

## Background

In recent decades complementary and alternative medicine (CAM) has been widely used in countries around the world, including Norway [1–3], Scandinavia [2, 4], Europe [5] and elsewhere [6–9]. In recent years the utilization of CAM appears to have stabilized [10]. The self-reported use of CAM varies from 10% [5] to 76% worldwide [11]. When restricted to visits to a CAM provider, the use varies between 2 to 49% [11]. These large variations in self-reported use are mostly due to differences in how CAM is understood, different timeframes in which CAM use is reported, and differences in national regulations [12].

The concept of CAM implies a distinction between complementary medicine, i.e., services, products, or practices used alongside conventional medicine, and alternative medicine, i.e., services, products, or practices used instead of conventional medicine [13]. Subcategories of health care-seeking behaviour fall under the umbrella of CAM, such as visits to CAM providers; use of herbal medicines and dietary supplements, and different types of self-help practices [14]. Consequently, the prevalence of CAM use depends on what types of services, products, and self-help practices are included in the definition of CAM.

To overcome reporting issues and to improve comparison between countries, a unified investigation tool to measure the use of CAM (I-CAM-Q) was developed in 2006. The development of the instrument was initiated by of the Norwegian National Research Center in Complementary and Alternative Medicine (NAFKAM), in cooperation with a group of international experts in the field. The questionnaire was presented in a publication in 2009, with instructions for translation and cultural adaptation [14]. From 2012 to present, the questionnaire has been translated and adapted to many different countries and languages [15–20]. The I-CAM-Q instrument has been applied worldwide such as in France [15], Germany [20], Iran [16], Saudi Arabia [21], US [22, 23], Argentina [24], Japan [25, 26], Taiwan [17], Korea [18], Cambodia [27] and Australia [28]. In 2012 Eardley et al. published a paper with the aim to “generate preliminary evidence concerning the face validity, acceptability and basic characteristics of the I-CAM-Q across different populations”. They concluded that the I-CAM-Q had low face validity and low acceptability and therefore likely produced biased estimates of CAM use in the populations they studied (England, Romania, Italy, The Netherlands and Spain) [29].

Opheim et al. [20], applied the I-CAM-Q instrument to map the use of CAM in Norwegian patients who suffered from inflammatory bowel disease. They found that 49% of the patients had used some CAM modalities within the past 12 months. CAM services were utilized by 27% of the patients, 21% reported use of CAM products, and 28% had used self-help practices [30]. In 2017, Wemrell et al. [19] applied the I-CAM-Q instrument when investigating the utilization of CAM in Sweden. The researchers found that 71% of the respondents had used CAM in the past year. A total of 33% had visited CAM providers; 53% had used natural remedies, herbal medicine, or nutritional supplements; and 32% reported having used self-help practices [19].

Despite the fact that CAM in Norway is defined as “health-related treatment which is practiced outside the established health services and which is not practiced by authorized health personnel” [31], the treatment is still considered as CAM when practiced by authorized health personnel if the methods used are mainly used outside the national health care service [31]. In 2015, Jacobsen et al. found that 64% of the hospitals offered one or several modalities defined as CAM [32]. Also, authorized medical personnel working outside the hospitals offered CAM to some extent. Most commonly offered modality is acupuncture, which is often provided in conjunction with physiotherapeutic treatment [33].

The conventional healthcare system in Norway is characterized by the Nordic welfare model with universal rights and equality based on governmental re-distribution of taxation revenue. Treatment offered within conventional healthcare is funded by the health authorities and free of charge for the patients or co paid with a small fee [34]. CAM offered outside the national health care service is fully paid for out of pocket. CAM providers offer their services mostly complementary to conventional treatment [35]. With few exceptions, and mainly in specific disease groups, CAM use in Norway has been reported as frequency and associations of total CAM use rather than for specific therapies [3, 10, 36]. We have therefore limited knowledge of which therapies, natural products, and self- help techniques that are used in Norway, and for what purpose people use the therapies. Neither do we know how helpful they find these therapies.

### The aim

Based on the adapted I-CAM-Q instrument, we (1) described the proportion using specific CAM modalities among a representative sample of participants above 16 years of age in Norway, and (2) identified the self-reported purposes and perceived helpfulness of these modalities.

## Methods

### Sampling and recruitment

In order to generalize from a random sample and avoid sampling errors or biases, a random sample needs to be of adequate size [37]. With a margin of error of 5%, a confidence level of 95%, and a heterogeneity of 50%, we needed a minimum sample of *n* = 385 to represent the Norwegian population of 5,328,000 inhabitants. As increased sample size is associated with decreased sampling error and is more likely to represent the population [38], the sample size was set to *n* = 2000. 

**Fig. 1:**
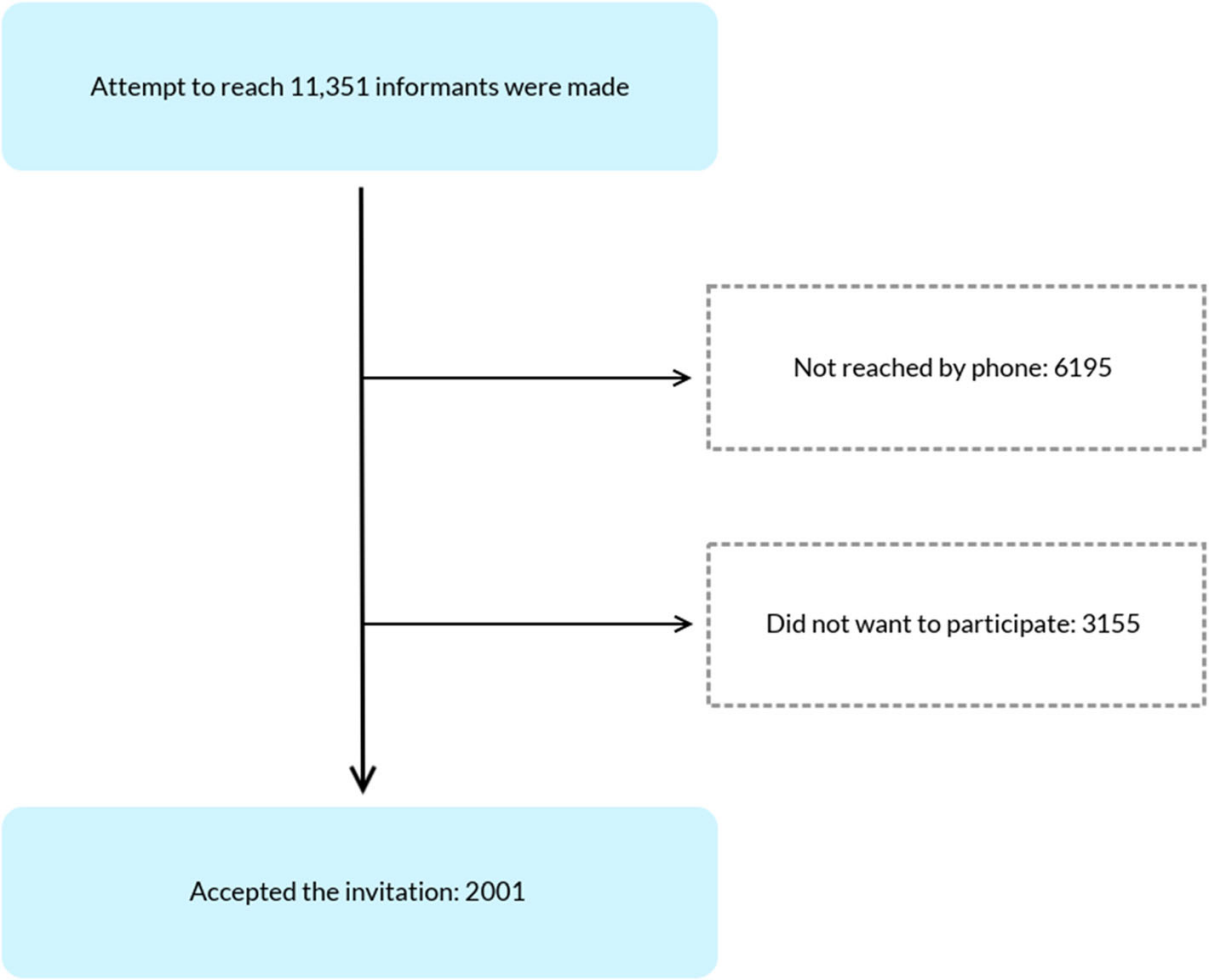
Flow chart of the included participants

A national survey based on computer-assisted telephone interviews was conducted between January 21 and February 3, 2019, in collaboration with the marketing research company Ipsos A/S [39]. The sample was drawn from Norwegians aged 16 and above living in private households with a landline telephone or a cell phone using random quota sampling. Quotas by age, sex, and region of residence were established to obtain a sample representative of the adult population of Norway. When calling a landline number, the interviewer asked for the person in the household who was 16 years of age or older with the most recent birthday. When calling a cell phone number, the person answering the phone was interviewed directly with the following request: *“Good evening, my name is …*. *and I’m calling from Ipsos MMI. We are conducting an important survey on health and consumption. In connection with that, I would like to ask you some questions. Is it okay?”*. Up to 7 attempts were made to reach the selected person. *N* = 6195 were unreachable after 7 calling attempts (Fig. 1).

Individuals who were reached and refused participation (*n* = 3155) were considered non-responders, leading to a response rate of 38.8%. The final sample contained 2001 individuals, 166 recruited on the basis of landline numbers and 1835 on the basis of cell phone numbers.

### Survey instrument

A modified Norwegian version of the I-CAM-Q instrument was used when interviewing the participants (attached as supplemental material, I-CAM-QN). The questionnaire included questions about CAM modalities such as CAM modalities provided by CAM providers and physicians in addition to self-help practices; natural remedies; herbal medicine including vitamins/minerals, homeopathic remedies, and other supplements (see Tables 2, 3, 4 and 5 in the Result section). The I-CAM-Q was translated directly by the research group regarding all questions and therapies in the original I-CAM-Q. Also the therapies were translated and placed in the same order as in the original I-CAM-Q. Regarding visits to health providers were the following providers added: Reflexologist, kinesiologist, massage therapist, naparapath, osteopath and cupping therapist. For visits to physicians the following therapies were added: reflexology, kinesiology, massage, naparapathy, osteopathy and cupping. Regarding self-help practices, mindfulness, lightning process, and neurolinguistic programming (NLP) were added.

Other data collected at the time of interview were sex, region of residence in Norway, age, household income, and highest level of education completed.

### Measures

#### Measures of personal characteristics

Household income was collected using the following categories (NOK < 100,000, 100,000-199,000, 200,000-299,000, 300,000-399,000. 400,000-499,000, 500,000-599,000, 600,000-799,000, 800,000-999,000, 1000,000–1500,000 and more than NOK 1500,000). These were collapsed into a measure of household income of low (<NOK400,000), middle (NOK 400,000-799,000) and high (NOK 800,000 or more).

Level of education was recorded using six values: primary school up to 8 years; primary school up to 10 years; secondary school; college/university less than 4 years; and college/university 4 years or more. These were merged into a measure with four categories (primary school; secondary school; college/university less than 4 years; and college/university 4 years or more). Due to changes in number of years of mandatory school in Norway, varying over the time from 7, through 9 and now 10 years, participants might have different number of years included in their primary school background.

Age was obtained as an open question and assessed as a continuous variable. In the analysis this was in addition categorized into a measure of age with three levels (16–29 years; 30–59 years; and 60 year or more).

Other personal characteristics included sex (female, male) and residence (merged into the Norwegian regions South-East, South, West, Mid (Trøndelag), and North.

#### CAM therapies provided by CAM providers and physicians

The measure *use of CAM received from providers* had two response options CAM received from CAM providers and CAM received from physicians. Some modalities that are considered CAM internationally, are not considered CAM in Norway. In the tables these modalities are marked with a *.

The participants were asked whether they had visited a provider within the last 12 months. For those who responded *yes*, they were asked the number of visits within the last 3 months, purpose of visit (acute illness lasting less than a month, long-term illness lasting more than a month, to improve wellbeing, and other reasons), and whether it was regarded as helpfulness with the response options very helpful, somewhat helpful, not helpful, and don’t know.

#### CAM self-help practices

Dichotomous measures assessed the *use of self-help practices* used during the last 12 months. The practices queried were meditation, yoga, qigong, tai chi, relaxation, visualization, traditional healing rituals, prayer for own health, mindfulness, lightning process, neuro-linguistic programming (NLP), and other. For each practice used, respondents reported number of visits within the last 3 months, purpose of visit (acute illness lasting less than a month, long-term illness lasting more than a month, to improve wellbeing, and other reasons), and whether it was regarded as helpfulness with the response options very helpful, somewhat helpful, not helpful, and don’t know.

#### Herbal medicine and dietary supplements

An open-ended question measured the use of herbal medicine and dietary supplements. The respondents had to state the name of the product(s) they have used within the last 12 months in the following categories: herbs/herbal medicine, vitamins/minerals, homeopathic remedies, and other supplements. For each product used, the respondent was asked if they used it at present, reason for use, and helpfulness. In the analysis these modalities were collapsed into four groups (h*erbs/herbal medicine*, *vitamins/minerals*, *homeopathic remedies*, and *other supplements*)*.* Many of the products were misclassified by the respondents (for example an herb was reported as a vitamin/mineral). The products were therefore reclassified into the right category by the first and the last author. Due to these misclassifications, the responses *reason for use* and *helpfulness* became inaccurate and could not be used in further analysis.

#### Over-all use of CAM

Over-all-CAM use was measured by calculating the total number of CAM users reported, combining the variables CAM modalities provided by CAM providers and physicians, natural remedies considered as CAM, and CAM self-help practices.

### Statistics

Descriptive statistics were carried out using Statistical Package for Social Sciences (SPSS) v. 26.0. Pearson’s Chi-square test and Independent-Samples T-tests were used to identify differences in sociodemographic (age, education level, household income), between men and women as well as between users and non-users of CAM. For all analyses the data were weighted to represent the Norwegian population in regard to age, sex, education and region. No further adjustments were made. Table 1 shows how the collected sample differs from the weighted sample.

**Table 1:** Basic characteristics of the participants

	The total sample %		Users of CAM^**1**^%		Non-users of CAM%		***p***-value	
	Unweighted sample	Weighted sample	Unweighted sample	Weighted sample	Unweighted sample	Weighted sample	Unweighted sample	Weighted sample
**Total CAM use**^a^	100	**100**	61.9	**62.2**	38.1	**37.8**		
**Sex**							< 0.001*	< 0.001*
Men	51.4	**50.2**	51.5	**51.6**	48.5	**48.4**		
Women	48.6	**49.8**	72.8	**72.9**	27.2	**27.1**		
**Age**								
Mean age (SD)	**46.80 (18.79)**	**47.31 (18.74)**	45.94 (18.71)	**46.34 (18.70)**	48.19 (18.85)	**48.91 (18.72)**	0.009**	0.003**
**Age groups**								
16–29 years	22.7	**21.9**	64.6	**65.5**	35.4	**34.5**	0.037*	< 0.029*
30–59 years	50.0	**50.3**	63.0	**63.2**	37.0	**36.8**		
60 year or more	27.2	**27.9**	57.4	**57.8**	42.6	**42.2**		
**Household Income**							0.040*	< 0.035*
Low	15.9	**15.8**	69.6	**70.2**	30.4	**29.8**		
Middle	32.3	**32.4**	60.4	**60.9**	39.6	**39.1**		
High	51.8	**51.8**	62.1	**62.1**	37.9	**37.9**		
**Years of Education**							< 0.001*	< 0.001*
Primary school	9.5	**9.8**	56.3	**56.9**	43.7	**43.1**		
Secondary school	38.0	**37.6**	57.2	**57.8**	42.8	**42.2**		
College/university less than 4 years	31.4	**31.5**	68.9	**68.8**	31.1	**31.2**		
College/university 4 years or more	21.0	**21.1**	62.4	**62.6**	37.6	**37.4**		
**Region**							0.221*	< 0.234*
South East	50.1	**50.5**	64.1	**64.5**	35.9	**35.5**		
South	5.5	**5.6**	56.0	**56.6**	44.0	**43.4**		
West	27.0	**26.3**	59.3	**59.8**	40.7	**40.2**		
Mid (Trøndelag)	8.3	**8.2**	63.3	**62.4**	36.7	**37.6**		
North	8.8	**9.3**	59.9	**59.7**	40.1	**40.3**		

^a^ Users of CAM include participants who received CAM from any provider, who used CAM self-help techniques or reported intake of CAM over the counter remedies; * Pearson Chi-square test, ** Independent-Samples T-test

## Results

### Basic characteristics of the participants

Most of the participants in this study were between 30 and 59 years old, had high household income, university education, and lived in the South-East part of Norway around the capital Oslo. The typical user of CAM was young and female, with a university degree (up to 4 years) and lower income, living in the South-Eastern part of Norway, the area around the capital (Table 1).

### Use of CAM

More than sixty percent (62.2%) of respondents reported using any CAM during a 12 months period (Table 1). The CAM modalities reported are presented in Tables 2, 3, 4 and 5. The most commonly used CAM modality was *natural remedies* including herbal medicines, homeopathic remedies and dietary supplements (Table 5).

**Table 2:** Any treatments received from any providers during the last 12 months

			Motivation %					Mean number of visits during last 3 months (SD)
	Used last 12 months (%) Total (women) men	*p*-value sex	Acute illness	Long-term illness	Improvement of wellbeing	Other	Helpfulness % (Very or somewhat)	
Physicians^a^	76.1 (84.1) 68.1	< 0.001	25.5	38.6	6.0	29.9	95.2	4.39 (5.74)
Chiropractors^a^	10.2 (11.3) 9.1	0.093	33.2	46.1	4.8	15.9	93.0	5.60 (6.38)
Homeopath	0.8 (1.0) 0.7	0.456	7.1	69.3	5.4	18.1	94.1	4.48 (3.41)
Acupuncturist	2.7 (3.4) 2.0	0.050	11.0	49.0	13.0	27.0	85.4	6.24 (6.62)
Herbalist	0.6 (0.9) 0.4	0.160	7.6	22.4	39.7	30.3	84.8	3.57 (3.54)
Healer	0.9 (1.3) 0.6	0.103	0.0	31.3	26.3	42.3	94.3	6.77 (11.33)
Traditional healer	0.2 (0.4) 0.1	0.177	0.0	38.0	20.8	41.1	100	3.71 (3.73)
Reflexologist	1.4 (2.1) 0.7	0.007	3.2	32.6	49.1	15.1	92.0	3.76 (4.31)
Kinesiologist	0.2 (0.3) 0.2	0.649	0.0	38.7	15.9	45.3	100	4.15 (3.09)
Massage therapist	7.4 (10.6) 4.3	< 0.001	7.8	22.4	55.3	14.4	95.0	4.74 (10.75)
Naprapath^b^	3.1 (3.3) 3.0	0.680	23.2	55.4	6.3	15.0	91.3	4.68 (3.91)
Osteopath	1.6 (2.3) 0.9	0.012	6.7	74.0	3.3	16.0	92.5	5.22 (4.63)
Cupping therapist	0.7 (0.9) 0.5	0.278	7.3	43.1	19.9	29.8	94.2	6.49 (6.51)
Other	0.5 (0.7) 0.3	0.201	14.6	56.1	4.6	24.7	94.8	11.95 (16.66)
Over-all use of CAM providers	14.7 (19.4) 10.1	< 0.001						

^a^ Authorised health care providers in Norway and therefore not considered as CAM providers. ^b^ Naprapathy is a system of specific examination, diagnostics, manual treatment and rehabilitation of pain and dysfunction in the neuro-musculoskeletal system

**Table 3:** CAM treatments received from physicians last 12 months

		Motivation %				
	Used by % within the last 12 months	Acute illness	Long-term illness	Improvement of wellbeing	Other	Helpfulness % (Very or somewhat)
Manipulation^a^	0.2	0.0	66.9	0.0	33.1	100
Homeopathy	0.2	0.0	64.1	0.0	35.9	100
Acupuncture	0.6	14.2	43.3	18.0	24.5	84.1
Herbal medicine	0.04	0.0	0.0	100	0	–
Healing	0.1	0.0	69.2	0.0	30.8	69.2
Traditional healing	0.04	0.0	0.0	100	0.0	100
Reflexology	0.04	0.0	100	0.0	0.0	100
Kinesiology	0	–	–	–	–	–
Massage^a^	0.6	38.1	22.5	13.3	26.1	92.2
Naparapathy	0.2	0.0	100	0.0	0.0	76.4
Osteopathy	0.1	0.0	100	0.0	0.0	100
Cupping	0.1	0.0	0.0	0.0	100	100
Other	0.1	8.9	61.5	8.8	20.9	100

^a^ Not consider as CAM when performed by physicians

**Table 4:** Self-help practices used last 12 months

			Motivation %					Number of times practiced last 3 months (SD)
	Used % Total (women) men	*p*-value sex**	Acute illness	Long-term illness	Improvement of wellbeing	Other	Helpfulness % (Very or somewhat)	
Meditation	9.4 (11.1) 7.7	0.008	1.1	19.9	57.1	21.8	95.1	25.79 (31.34)
Yoga	14.8 (24.1) 5.6	< 0.001	0.9	14.7	57.2	27.2	95.3	12.71 (20.18)
Qigong	0.9 (1.5) 0.3	0.004	0.0	25.5	31.4	43.2	93.7	15.43 (25.21)
Tai Chi	0.9 (1.5) 0.3	0.017	0.0	28.9	13.7	57.3	94.0	20.86 (31.51)
Relaxation	9.8 (14.2) 5.4	< 0.001	2.5	28.9	46.1	22.5	97.7	29.15 (33.46)
Visualization	3.8 (4.8) 2.9	0.025	2.7	15.8	33.9	47.6	96.0	29.34 (33.44)
Participation in traditional healing rituals	0.3 (0.2) 0.4	0.418	0.0	13.7	0.0	86.3	84.4	7.96 (4.14)
Prayer for own health*	4.2 (5.0) 3.3	0.053	7.2	22.6	36.6	33.6	95.8	46.58 (40.31)
Mindfullness	5.5 (7.4) 3.6	< 0.001	0.0	22.9	47.7	29.3	95.1	29.13 (34.08)
Lightning process	0.0		–	–	–	–	–	–
NLP	0.4 (0.3) 0.6	0.322	0.0	38.3	12.1	49.6	100	39.98 (44.84)
Other	1.5 (2.0) 1.0	0.063	0.0	20.8	50.1	29.1	100	27.01 (26.70)
**Over-all use of self-help practises**	29.5 (39.0) 20.0	< 0.001						
**Over-all use of CAM self-help practises**	29.1 (38.9) 19.3	< 0.001						

* Not consider as CAM in Norway; ** Pearson Chi-square test

**Table 5:** Natural remedies used last 12 months

Natural remedies	Total %	Women %	Men %	*p*-value**
Herbs/Herbal Medicine	7.2	9.4	5.0	< 0.001
Vitamins/Minerals	51.1	60.2	42.1	< 0.001
Vitamin/Minerals excluding multivitamins* used by 16%	42.6	51.6	33.8	< 0.001
Homeopatic remedies	0.3	0.4	0.3	0.698
Other supplements	33.7	37.9	29.5	< 0.001
Other supplements excluding cod liver oil* (19%) and Omega-3 supplement* (11%)	7.8	9.4	6.1	0.005
Over-all use of natural remedies	68.1	76.2	60.1	< 0.001
Over-all use of CAM natural remedies	47.7	56.9	38.6	< 0.001

* Not consider as CAM in Norway; ** Pearson Chi-square test

### CAM treatments received from providers

A total of 15.4% of the respondents had received CAM from a CAM provider or from a physician during the last 12 months (Tables 2 and 3). The majority received treatment from CAM providers (14.7%, Table 2) fewer from physicians (1.2%, Table 3). The five most commonly used modalities were manipulation (including chiropractic, naprapathy and osteopathy), (14%), massage (8%), acupuncture (3.1%), reflexology (1.4%), and healing (1.2%) (Tables 2 and 3).

### Visits to CAM providers

The respondents consulted the following top three providers: massage therapists (7.4%), naprapaths (3.1%), and acupuncturists (2.7%, Table 2). The majority of the participants found the treatment helpful (85–100%, Table 2). They used most modalities for long term illnesses. However, massage, reflexology and herbs were mainly used for improving well-being. Healing and kinesiology, on the other hand, were used for other purposes, such as psychological complaints (Table 2). Men and women consulted most providers to a similar degree. However women visited osteopaths, massage therapists, and reflexologists more frequently than men (*p* < 0.05, Table 2). Most of the participants who had visited a CAM provider had also visited a physicians in the same period (82.3%).

### CAM treatments received from physicians

The majority (76.1%) of the participants had visited a physician during the last year, but only 1.2% had received CAM treatments from their physician (Table 3). The most commonly provided therapies were massage therapy (0.6%) and acupuncture (0.6%) followed by manipulation (including naprapathy and osteopathy, 0.4%), hands on healing (0.2%), and cupping (0.1%). The therapies were mostly used for long-term illness and the participants found the treatment helpful (69.2-100%, Table 3).

### Self-help practices

Almost one third (29.5%) of the respondents used at least one self-help modality. When prayer was excluded (the modality is not considered CAM in Norway), the use decreased to 29.1%. The modalities most used were yoga (14.8%), meditation/ mindfulness (12.2%), relaxation (9.8%), prayer (4.2%), and visualization (3.8%, Table 4). How often the modalities were practiced during a 3 month period varied from 7.96 times (participation in traditional healing rituals) to 39.98 times (NLP). The majority used self-help practices to improve well-being or for other reasons like fitness, mental training, health prevention, and for spiritual- and psychological purposes (Table 5). Most respondents found these modalities helpful (84–100%). Generally, women practiced self-help modalities more than men (*p* < 0.001). However, use of NLP, (*p* = 0.322), traditional healing rituals (*p* = 0.418), and prayer for own health (*p* = 0.053) were similar for men and women (Table 4).

### Natural remedies

Two third (68.1%) of the respondents had used natural remedies (Table 5). The most commonly used remedies were cod-liver oil (19%), D-vitamin (18%), multivitamins (16%), omega-3 fatty acids (11%), C-vitamin (10%), magnesium (8%), B-vitamin (8%), iron (4%), calcium (3%), and blueberry extract (1%). When remedies that are in traditional use in Norway and therefore not considered CAM (multivitamins, cod-liver oil, and Omega-3 fatty acids) were excluded, the use of natural remedies decreased to 47.7% (Table 5).

## Discussion

A total of 62.2% of the participants reported to have used CAM within the last 12 months. Most participants had used CAM natural remedies (47.7%), followed by CAM self-help practices (29.1%) and CAM modalities received by CAM providers (14.7%). A minority had received CAM modalities from physicians (1.2%). The typical users of CAM were young women with a lower university degree. Mean number of visits to CAM providers ranged from 3.57 times (herbalist) to 6.77 times (healer). Most of the participants found CAM helpful.

Over-all CAM use of 62.2% found in the present study was a higher proportion compared to the first Norwegian study based on the I-CAM-QN by Opheim et al. [30] where 49% of the participants reported to have used some type of CAM. However, Opheim et al. reported higher use of CAM providers (27% vs 14.7%). The reason for this discrepancy may be that the present study investigated a healthy population while Opheim et al. investigated a patient group suffering from inflammatory bowel disease. The use of CAM self-help practices were, however, similar in the two populations (29.1% vs 28%). Use of CAM natural remedies was substantially higher in the present study (47.7% vs. 21%). The main reason may be that Opheim et al. did not consider any vitamins and minerals as CAM in the analyses. When we excluded vitamins and minerals from the analyses, we found a 14.2% prevalence of CAM natural remedies with a total CAM prevalence of 42.9%, which is in line with their findings of 49% CAM use.

Higher use of CAM was also found in the present study compared to another Norwegian study, the NAFKAM study [40], that investigated a similar population 2 months earlier. We found twice as many CAM users compared to the NAFKAM study (62.2% vs. 37%). Despite the fact that our participants reported higher overall use of CAM, the NAFKAM study reported a higher number of participants who received CAM modalities from providers (23% vs. 15.4%). The main reason for this discrepancy may be that the NAFKAM study mapped modalities used (acupuncture, massage etc. given by providers) while we mapped visits to specific providers (acupuncturist, massage therapist etc.). The difference between use of CAM modalities and visits to CAM providers has been shown to give different proportions of CAM users in Norway [41], as a CAM provider can offer more than one therapy in the same session and therefore increase the number of therapies and not visits to providers. Further, the therapies can be received from people who do not identify themselves as CAM providers (like physiotherapists, nurses etc.), though they offer a CAM modality (like acupuncture) during their treatment. The NAFKAM study also include CAM modalities received from other health care providers than CAM therapists and physicians [40]. Use of CAM natural products and CAM self-help-practices were however higher in the present study compared to the NAFKAM study (29.1% self-help-techniques vs. 17%, and 47.7% use of CAM natural products vs. 10%). The reason for the higher use of CAM natural products in the present study is suspected to be due to the exclusion of vitamins and minerals as CAM natural products in the NAFKAM study. As shown above the prevalence of CAM natural products in the present study decreased from 47.7 to 14.2% when vitamins and minerals were excluded. The most plausible explanation of the higher use of CAM self-help -practices in the present study seems to be the comprehensive list of modalities listed as response options in our study in contrast to the single question used in the NAFKAM study [40]. The list is likely to work as a reminder for practices used, and a clarification of how to understand what to consider as CAM self-help practises.

Higher use of CAM was also found in the present study compared to a regional study in the county of Tromsø conducted in 2015–2016 [42]. In this study, Kristoffersen at al found 30.1% over-all-CAM use; 13.6% reported to have seen a CAM provider, 17% had used CAM natural remedies and 10.2% had used CAM self-help practices. The use of CAM providers was similar, while use of CAM natural remedies and CAM self-help practices were higher in the present study. Again, more specified lists of CAM modalities may give a higher response rate than just giving the participants one question asking about if they have used CAM natural remedies/CAM self-help practices or not.

Despite higher reported use of CAM in the present study compared to other Norwegian studies, our prevalence of CAM users were somewhat lower (62.2% vs. 71%) than found in a Swedish study using I-CAM-Q conducted by Wemrell et al. [19]. Small differences were reported regarding CAM natural remedies and CAM self-help - practices, visits to CAM providers were, however twice as high in Sweden than in Norway (33% vs. 14.7%). The high use of CAM providers may be partly due to the inclusion of chiropractors as CAM providers, in addition to higher prevalence of the use of massage. If we included chiropractors in our definition and analysis of CAM providers, we reached a prevalence of 22.2% for the use of a CAM provider.

Higher use of CAM was also found in a German study where the I-CAM-Q questionnaire was used. Krug et al. [43] reported that 78% of a breast cancer sample have used CAM. A total of 26% of the participants had visited a CAM provider while 28% had received CAM from their physician. Herbal medicine and dietary supplements were used by 60% of the population. A higher prevalence (55%) of self-help practices were also found in the German study. The higher use of CAM in the German study can be explained by the high proportion of CAM modalities received from physicians and a much higher use of self-help practises. One possible explanation of this high prevalence of CAM may be that the investigation was performed among breast cancer patients and that CAM modalities are integrated in conventional health care in Germany to a higher degree than in Norway [43].

The most used health care received from providers were musculoskeletal therapies like massage, manipulation, chiropractic, naprapathy, and osteopathy. These modalities were used by 12% of the men and 16% of the women. This correlates well with the number of people in Norway who suffer from musculoskeletal disorders (18% of the men and 27% of the women) [44], indicating that these modalities may be commonly used by people with musculoskeletal disorders. The most commonly reported musculoskeletal disorders in Norway are lower back and neck pain [44].

In general, percentage of participants who received CAM from physicians was low. This was also the case in the Swedish study [19]. This underlines the impression that CAM is mostly offered outside public health care in the Scandinavian countries, which is in line with the Norwegian definition of CAM [31]. This is not in accordance with the situation in other European countries. In Germany, 68% of the frequent users of CAM reported having received CAM from a physician [20]. These major differences can be explained as cultural differences and CAM regulations [45]. While only a very few physicians in Norway report having training in CAM [46], the situation is quite the opposite in Germany where more than 67,000 physicians have qualified training in CAM and 60% provide CAM to their patients [43]. In addition, the regulation of CAM differs widely in Europe. While CAM can be practiced by both authorized health care and lay trained providers in the Nordic countries, UK, Germany and the Netherlands, only authorized health care providers are allowed to practice CAM in southern and eastern European countries [45].

Most of the participants in this study found CAM to be helpful/very helpful regardless of CAM modality. This is in accordance with the findings in Sweden [19] and Germany [20]. Every CAM modality demands an effort from the user such as money spent, time and time to practice etc. Consequently, we argue, that if people perceive the modality to be useless, they would terminate the modality.

This study must be understood in light of its limitations, and a main limitation is the low response rate of 39% which may challenge the generalizability of the findings. The generalizability increases, however, if the sample is representative for the target population. The population selected for this study was first stratified by region, age, and sex. All included participants were, in addition, weighted in regard to age, sex, educational level and region giving a nationally representative sample in regards to these factors. This increases the generalisability of the findings presented in this paper. When the participants were contacted by IPSOS, the study was not presented as a CAM study. We have therefore no reason to believe that participants who were pro-CAM were more likely to accept the invitation and participate in the study. The study was, however, presented as a health related study so the non-responders might have a lower interest in health issues in general than the responders. As the data was collected through telephone interviews we believe, however, that the non-responders mainly consisted of people who did not have time to take part in the study at the time they were contacted by the interviewer, and people who, in general, do not participate in telephone surveys.

The validity of self-reported data can also be questioned. The agreement, however, between self-reported data and registered health care use is generally high [47]. The I-CAM-Q has, however, been shown to have low face validity and low acceptability in five EU countries when a self-administrated, paper questionnaire was used, [29]. We believe that using personal interviewers capable of answering questions and clarify questions for the respondents upon request, has reduced this bias. The 100% anonymity of the respondents in this survey may increase the validity of sensitive information such as health and health care visits [48]. Recall bias, could on the other hand, be a threat to the validity of the findings as the use of CAM was asked for in a 12 months period. In particularly number of time the different CAM modalities were used could be difficult to remember even though this was asked for only during a 3 month period.

### Implementation of the findings

In this study we have for the first time presented use of specific CAM therapies in all areas of CAM in Norway (CAM providers, CAM products and CAM self-care) and further described specifically for each therapy why they are used and how helpful the modality was to the patients. This will increase health care providers’ general knowledge of CAM use among their patients, and further help them to increase their knowledge of the most commonly used therapies and how they might interact with treatment they offer themselves to avoid negative interactions between CAM use and conventional care. As CAM users are people who express health care needs beyond ordinary public health care in Norway, health care providers need to identify them to be able to provide them with patient-centred health care in an open-minded, non-judicial way [36]. To be able to do this, earlier research has urged the need for more detailed information of the users and their reason for using CAM [36].

### Further research

As the present study describes CAM use in a population without specific health problems, further research is needed in disease-specific populations to compare CAM use in a healthy population with different disease specific populations. Different from in many other countries, previous research in Norway has shown similar use of CAM in cancer populations compared to the general population. In further research the I-CAM-Q should be used to see if these similarities remain when a more specific questionnaire is used.

## Conclusion

This study confirms that CAM is used by a considerable part of the Norwegian population, and that the prevalence of CAM use increases when specific modalities are listed as response options in the questionnaire as a reminder to the respondents. The CAM modalities used are mainly received from CAM providers operating outside public health care or administered by the participants themselves. 

## Supplementary Information

**Additional file 1.**

## Data Availability

The dataset this paper has been based on has not been deposited in any repository. All dataset and materials are available from the corresponding author upon reasonable request.

## Abbreviations

CAM: Complementary and Alternative Medicine
NLP: Neuro-Linguistic Programming
