# Supplementary material for: Use of complementary and alternative medicine in Norway: a cross-sectional survey with a modified Norwegian version of the international questionnaire to measure use of complementary and alternative medicine (I-CAM-QN)
Source: BMC Complement Med Ther. 2021 Mar 16;21:93. doi: 10.1186/s12906-021-03258-6 (PMC7962303; doi:10.1186/s12906-021-03258-6)
Supplement: Supplementary file 1 — Additional file 1. [file 12906_2021_3258_MOESM1_ESM.docx]

***1. Besøk hos behandlere:*** Forskjellige alternative behandlere og ansatte innen skolemedisin kan ta seg av helseproblemer.

| Har du vært hos en eller flere av følgende behandlere de siste 12 månedene? | Ja  Nei  Antall ganger du var hos denne behandleren de siste 3 månedene? | Angi *hovedårsaken* til at du *sist* gikk til behandleren  (Kun ett kryss). | | | | Hvor nyttig var det for deg å gå til denne behandleren?  (Kun ett kryss) |
| --- | --- | --- | --- | --- | --- | --- |
|  |  | For en akutt sykdom/tilstand med varighet kortere enn én måned. | For å behandle et langvarig helseproblem (varighet lenger enn én måned) eller symptomer knyttet til dette | For økt velvære | Annet  (Vennligst spesifiser den andre årsaken) |  |
|  |  |  |  |  |  | Veldig nyttig  Litt nyttig  Ikke nyttig  Vet ikke |
| **Lege** |  |  |  |  |  |  |
| **Kiropraktor** |  |  |  |  |  |  |
| **Homøopat** |  |  |  |  |  |  |
| **Akupunktør** |  |  |  |  |  |  |
| **Urtemedisiner** |  |  |  |  |  |  |
| **Healer** |  |  |  |  |  |  |
| **Håndspålegger / leser** |  |  |  |  |  |  |
| **Fotsoneterapeut** |  |  |  |  |  |  |
| **Kinesiolog** |  |  |  |  |  |  |
| **Massør** |  |  |  |  |  |  |
| **Naprapat** |  |  |  |  |  |  |
| **Osteopat** |  |  |  |  |  |  |
| **Kopping** |  |  |  |  |  |  |
| **Annen (skriv hvilken):**  **____________** |  |  |  |  |  |  |

***2. Alternative behandlinger mottatt fra leger***

Noen leger gir både alternativ og skolemedisinsk behandling.

| Har du fått noen av følgende alternative/ komplementære behandlingsformer av lege de siste 12 månedene? | Ja  Nei  Antall ganger du fikk denne behandlingen de siste 3 månedene | Angi *hovedårsaken* til at du *sist* fikk denne behandlingen (Kun ett kryss) | | | | Hvor nyttig var det å få denne behandlingen fra legen?  (kun ett kryss) |
| --- | --- | --- | --- | --- | --- | --- |
|  |  | For en akutt sykdom/tilstand med varighet kortere enn én måned | For å behandle et langvarig helseproblem (varighet lenger enn én måned) eller symptomer knyttet til dette | For økt velvære | Annet  (Vennligst spesifiser den andre årsaken) |  |
|  |  |  |  |  |  | Veldig nyttig  Litt nyttig  Ikke nyttig  Vet ikke |
| **Manipulasjon** |  |  |  |  |  |  |
| **Homøopati** |  |  |  |  |  |  |
| **Akupunktur** |  |  |  |  |  |  |
| **Urter** |  |  |  |  |  |  |
| **Healing** |  |  |  |  |  |  |
| **Handspåleggelse / lesing** |  |  |  |  |  |  |
| **Fotsoneterapi** |  |  |  |  |  |  |
| **Kinesiologi** |  |  |  |  |  |  |
| **Massasje** |  |  |  |  |  |  |
| **Naprapati** |  |  |  |  |  |  |
| **Osteopati** |  |  |  |  |  |  |
| **Kopping** |  |  |  |  |  |  |
| **Annen behandling (**skriv hvilken):  **____________** |  |  |  |  |  |  |

***3. Bruk av urtemedisin og kosttilskudd,*** inkludert tabletter, kapsler og flytende midler.

| For hver kategori nedenfor, skriv opp til tre produkter som du har brukt de siste 12 månedene. | Bruker du disse produktene nå? | Angi *hovedårsaken* til at du *sist* brukte dette produktet.  (Kun ett kryss). | | | | Hvor nyttig var dette produktet for deg?  (Kun *ett* kryss) |
| --- | --- | --- | --- | --- | --- | --- |
|  |  | For en akutt sykdom/tilstand med varighet kortere enn én måned | For å behandle et langvarig helseproblem (varighet lenger enn én måned) eller symptomer knyttet til dette | For økt velvære | Annet  (Vennligst spesifiser) |  |
|  |  |  |  |  |  | Veldig nyttig  Litt nyttig  Ikke nyttig  Vet ikke |
|  | Ja  Nei |  |  |  |  |  |
| **Urter/urtemedisin** | | | | | | |
| ________________ |  |  |  |  |  |  |
| ________________ |  |  |  |  |  |  |
| ________________ |  |  |  |  |  |  |
| **Vitaminer/mineraler** | | | | | | |
| ________________ |  |  |  |  |  |  |
| ________________ |  |  |  |  |  |  |
| ________________ |  |  |  |  |  |  |
| **Homøopatiske midler** | | | | | | |
| ________________ |  |  |  |  |  |  |
| ________________ |  |  |  |  |  |  |
| ________________ |  |  |  |  |  |  |
| **Andre tilskudd** | | | | | | |
| ________________ |  |  |  |  |  |  |
| ________________ |  |  |  |  |  |  |
| ________________ |  |  |  |  |  |  |

***4. Selvhjelpsteknikker***

| Har du brukt noen av følgende selvhjelpsteknikker de siste 12 månedene? | Ja  Nei  Antall ganger du har brukt denne teknikken de siste 3 månedene? | Angi *hovedårsaken* til at du *sist* brukte denne selvhjelpsteknikken. (Kun *ett* kryss) | | | | Hvor nyttig var denne selvhjelpsteknikken for deg?  (Kun *ett* kryss) |
| --- | --- | --- | --- | --- | --- | --- |
|  |  | For en akutt sykdom/tilstand med varighet kortere enn én måned | For å behandle et langvarig helseproblem (varighet lenger enn én måned) eller symptomer knyttet til dette | For økt velvære | Annet  (Vennligst spesifiser den andre årsaken) |  |
|  |  |  |  |  |  | Veldig nyttig  Litt nyttig  Ikke nyttig  Vet ikke |
| **Meditasjon** |  |  |  |  |  |  |
| **Yoga** |  |  |  |  |  |  |
| **Qigong** |  |  |  |  |  |  |
| **Tai Chi** |  |  |  |  |  |  |
| **Avspennigs-teknikker** |  |  |  |  |  |  |
| **Visualisering** |  |  |  |  |  |  |
| **Deltakelse i tradisjonell helbredelses-seremoni** |  |  |  |  |  |  |
| **Bønn for egen helse** |  |  |  |  |  |  |
| **Mindfullness** |  |  |  |  |  |  |
| **Lightning process** |  |  |  |  |  |  |
| **NLP (nevrolingv-istisk program-mering)** |  |  |  |  |  |  |
| **Annen (Skriv hvilken):**  **____________** |  |  |  |  |  |  |
